# Enhanced Susceptibility to Breast Cancer in Korean Women With Elevated Serum Gamma-Glutamyltransferase Levels: A Nationwide Population-Based Cohort Study

**DOI:** 10.3389/fonc.2021.668624

**Published:** 2021-05-27

**Authors:** Aeran Seol, Wenyu Wang, Se Ik Kim, Youngjin Han, In Sil Park, Juhwan Yoo, HyunA Jo, Kyung-Do Han, Yong Sang Song

**Affiliations:** ^1^ Department of Obstetrics and Gynecology, Seoul National University College of Medicine, Seoul, South Korea; ^2^ Cancer Research Institute, College of Medicine, Seoul National University, Seoul, South Korea; ^3^ Interdisciplinary Program in Cancer Biology, Seoul National University, Seoul, South Korea; ^4^ WCU Biomodulation, Department of Agricultural Biotechnology, Seoul National University, Seoul, South Korea; ^5^ Department of Agricultural Biotechnology, Seoul National University, Seoul, South Korea; ^6^ Department of Biomedicine & Health Science, The Catholic University of Korea, Seoul, South Korea; ^7^ Department of Statistics and Actuarial Science, Soongsil University, Seoul, South Korea

**Keywords:** breast cancer, gamma-glutamyltransferase (GGT), menopause, cancer incidence, biomarker, obesity, body mass index (BMI)

## Abstract

**Background:**

The incidence of breast cancer has been gradually increasing in Korea. Recently, the elevated level of serum gamma-glutamyltransferase (GGT) has emerged to be associated with the development and progression of some malignancies. This study aimed to determine the effect of serum GGT levels on the risk of developing breast cancer in Korean women.

**Methods:**

We used National Health Insurance Service Health Checkup data to examine the association between serum GGT levels and breast cancer development in Korean women. Women aged 40 years or older who participated in the Korean National Health Screening Examination between January 2009 and December 2009 and who did not develop any cancer within 1-year post examination were included in this analysis (n = 3,109,506). Cox proportional hazard regression analysis was conducted to calculate hazard ratios (HRs) with 95% confidence intervals (CIs).

**Results:**

Overall, an elevated serum GGT level was associated with the increased risk of developing breast cancer; compared to the Q1 group, the Q4 group showed a significantly increased breast cancer risk (HR: 1.120,95% CI: 1.08–1.162). Such a relationship was stronger in post-menopausal women than pre-menopausal women (HR: 1.173, 95% CI: 1.107–1.243; HR: 1.070, 95% CI:1.019–1.124). Women with a high GGT level (Q4) were also at an increased risk of developing carcinoma *in situ* (CIS) (HR: 1.114, 95% CI: 1.04–1.192). In post-menopausal women, the Q4 group also exhibited higher CIS risk (HR: 1.266, 95% CI: 1.132–1.416). However, no significant difference in the risk of developing CIS was observed between the Q1 and Q4 groups in pre-menopausal women. Further analysis revealed that obese, post-menopausal women with a high GGT level (Q4) were associated with an increased risk of developing breast cancer (HR: 1.214, 95% CI: 1.125–1.31) and CIS (HR: 1.348, 95% CI: 1.159–1.569).

**Conclusions:**

Our study results demonstrate that increased serum GGT level is a risk factor for developing breast cancer. The post-menopausal women group with obesity and elevated serum GGT level showed the highest incidence of breast cancer. Thus, serum GGT concentration could be a novel and potential risk factor for breast cancer. Further validation in different ethnic groups would be warranted.

## Introduction

Breast cancer is the most commonly diagnosed cancer in most countries and the leading cause of cancer death in over 100 countries (1, 2). There will be approximately 2.3 million new female breast cancer cases in 2020 (2). Incidence rates of breast cancer have been rising for most countries over the last decade (2). In the United States, breast cancer accounts for 30% of female cancers. Incidences and deaths were estimated to be 276,480 and 42,170 cases respectively in 2020. In addition, approximately 48,530 cases of ductal carcinoma *in situ* (DCIS) of the female breast are expected to be diagnosed in 2020 (3). In Korea, the incidence of breast cancer has been steadily increasing over the past 10 years. 25,452 new cases and 2,748 cancer deaths are expected to occur in Korea in 2020 (4).

Obesity is on the rise worldwide, and the importance is increasing as the association with cancer is revealed including breast cancer (5, 6). For breast cancer, several large studies have shown an increased risk of breast cancer with weight gain in post-menopausal women (7, 8). In Korea, nationwide studies have confirmed that obesity increases the risk of breast cancer in post-menopausal women (9). Notably, the peak age for breast cancer is between 40 and 50 years in Asian countries, whereas in the western countries, itis between 50 and 70 years (10). For instance, the peak age of breast cancer in the United States was 61 years while that was 47 years in Korea (10, 11). Therefore, the different epidemiologic background in different countries should not be neglected in cancer prevention.

Serum gamma-glutamyl transferase (GGT) is a serum marker for alcoholic liver disease, alcohol consumption, and bile duct condition (12, 13). In addition, it is an indicator of oxidative stress caused by factors including cardiovascular disease, diabetes, and stroke (14–16). GGT differs according to gender, ethnicity, and region. In Korea, the GGT level is an upward trend as in USA which might be related to changing environmental factors such as excessive iron intake, exposure to xenobiotics and impairment of cell membranes caused by nutrition (12). Recent studies have been reported that elevated GGT level is associated with the occurrence of several cancers such as prostate cancer, liver cancer including hepatocellular carcinoma (17–19). The association with elevated GGT levels in breast cancer has been studied in the UK, but a cut-off value for this study was not suggested (20).

In this study, we analyzed the association between GGT and breast cancer risk by using a large cohort data from the Korean insurance registry by including 3,109,506 women (≥40 years old) who underwent health check-ups in 2009.

## Methods

### Study Participants

**Figure 1:**
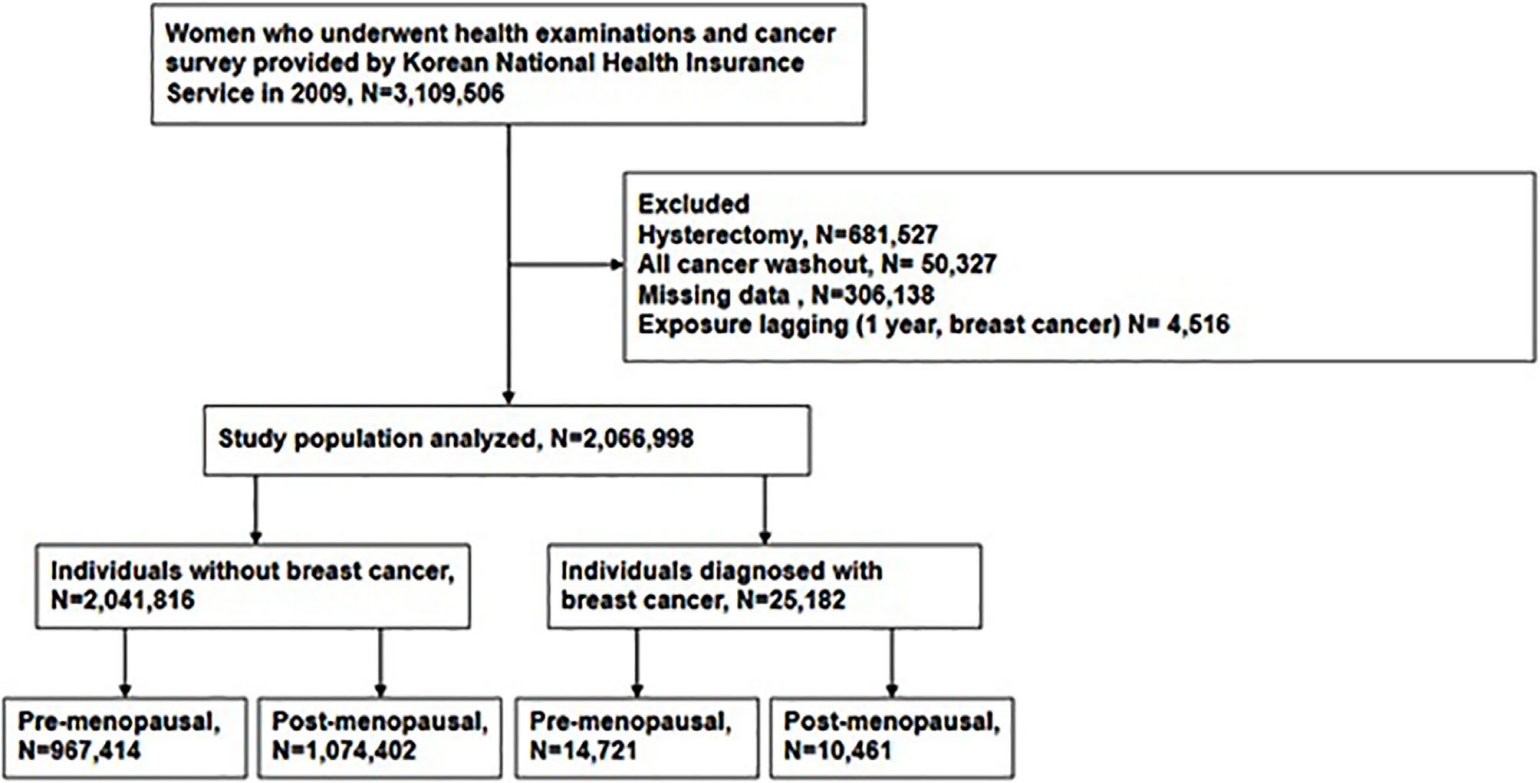
Flow chart depicting the design of the study participants by Korean National Health Insurances Service.

This study was a nationwide population-based cohort study using a Health Check-up database from the National Health Insurance Service (NHIS) of Korea. The NHIS is a single universal insurance service covering approximately 97% of the entire Korean population. In Korea, breast cancer screening tests are provided free of charge to all women over 40 years of age every 2 years. This test result is registered in the health check-up database of the NHIS. From the NHIS cohort, we identified women aged 40 years and older who received health examinations and completed the cancer-screening questionnaire between January 2009 and December 2009 (n = 3,109,506). Among them, we excluded women with the following conditions: (1) who reported that they had received a hysterectomy before; (2) who had been diagnosed with any cancer before health examination and had been registered with cancer registration code; or (3) those with insufficient data. If any cancer occurred within one year from the day of screening, it could have been caused by other cause than GGT. So we applied a 1-year lag period to minimize detection bias about the cancer diagnosis. Finally, 2,066,998 women were included in this analysis (**Figure 1**). The follow-up period is from the date of the NHIS Health Screening in 2009 to the onset of breast cancer or December 31, 2018, whichever comes first. In Korea, the national cancer registration project is in progress, when cancer or CIS is diagnosed, it is registered in the NHIS database. Women who met the inclusion criteria were followed up for breast cancer or CIS through the NHIS database.

### Data Collection

We retrieved detailed health check-up data, such as comorbidities, smoking and drinking status, and other lifestyle factors. Participants’ laboratory test results were also collected. Based on body mass index (BMI)at the time of health examination, we defined obese women as BMI ≥25.0 kg/m^2^ (21). Development of breast cancer and CIS was identified when participants made a documented visit to hospitals during the observation period with the registration code, V193 and the International Statistical Classification of Diseases, tenth revision (ICD-10) codes. The registration code of breast cancer is C50 and that of CIS is D05.

### Statistical Analysis

Incidences of breast cancer and CIS were calculated by dividing the number of events by 1000 person-years. Between the women who developed breast cancer (study group) and those who did not (control group), baseline demographic characteristics were compared using Student’s t test for continuous variables and chi-square test for categorical variables. Based on the quartiles of serum GGT levels, we stratified participants into each quartile group (Q1 to Q4) with an increasing GGT level.

Investigating the relationship between breast cancer and serum GGT levels, we used Cox proportional hazard regression models for multivariate analysis; the hazard ratios (HRs) and 95% confidence intervals (CIs) were calculated through cox proportional hazard regression using Q1 as a reference. The risk of breast cancer was evaluated in total women, pre- and post-menopausal groups, respectively. While none of the confounding factors were adjusted in Model 1, participants’ age was adjusted in Model 2. Model 3 adjusted for age, smoking status (3 levels), alcohol consumption (3 levels), regular physical activity and diabetes. Adding to this, Model 4 further adjusted for the number of parities, menopausal status, and age of menarche. For post-menopausal women, model 4 also included duration of hormonal replacement therapy as a confounder.

All statistical analyses were performed using R statistical software (version 3.4.4; R Foundation for Statistical Computing, Vienna, Austria; http://www.R-project.org). Statistical and SAS version 9.4 (SAS Institute, Cary, NC, USA) and a p value <0.05 was considered statistically significant.

## Results

### Baseline Characteristics of the Study Cohort

The mean follow-up periods was 8.37 years. Among the study cohort (n=2,066,998), 25,182 individuals (1.22%) developed breast cancer and assigned to the study group while 2,041,816 individuals (98.78%) were assigned to the control group (**Figure 1**). To analyze the association between GGT level and incidence of breast cancer in different menopausal status, each group was divided into pre- and post-menopausal subgroups (**Figure 1**).

Baseline characteristics of the two groups were displayed in **Tables 1** and **2** according to the menopausal status. Post-menopausal women who developed breast cancer showed higher proportions of diabetes mellitus, dyslipidemia, and smoking history compared to those who did not. Obesity was more prevalent in the study group than in the control group. Age was lower and the physical activity was higher in the breast cancer group. Serum GGT level is significantly higher in the post-menopausal breast cancer group (median 20.91 vs. 21.98, p<0.0001) while the GGT levels of pre-menopausal women did not differ between the two groups (median 17.29 vs. 17.46, p=0.1902). So we studied the effect of GGT on breast cancer incidence depending on whether menopause or not. We selected model 4 to adjust for other parameters that showed differences between the two groups.

**Table 1:** Baseline demographic and clinical data according to female cancer status in pre-menopausal women.

	Breast cancer		p-value
	No	Yes	
	(N = 967414, %)	(N = 14721, %)	
^*^Age (years)	45.04 ± 3.96	44.91 ± 3.92	<.0001
Smoking			0.4637
Never	918726 (94.97)	13947(94.74)	
Past smoker	15511(1.6)	246 (1.67)	
Current smoker	33177(3.43)	528 (3.59)	
Alcohol consumption			0.4158
No	692678(71.6)	10609 (72.07)	
Mild	263501(27.24)	3938 (26.75)	
Heavy	11235(1.16)	174 (1.18)	
Regular physical activity	166893 (17.25)	2503(17)	0.4283
Hypertension	138721(14.34)	2065 (14.03)	0.284
Diabetes mellitus	29311(3.03)	412(2.8)	0.1043
Dyslipidemia	110474(11.42)	1697(11.53)	0.682
^*^BMI (kg/m^2^)	23.24 ± 3.07	23.13 ± 3.02	<.0001
^*^Cholesterol	192.33 ± 39.16	192.07 ± 35.86	0.4253
^*^LDL	114.74 ± 71.38	113.69 ± 47.11	0.0746
^*^HDL	60.46 ± 35.65	60.56 ± 34.28	0.5615
^**^GGT	17.29(17.29, 17.31)	17.46(17.31, 17.61)	0.1902

^*^Parameter was indicated as mean +/- SD.

^**^Geometric means(95% C.I).

**Table 2:** Baseline demographic and clinical data according to female cancer status in post-menopausal women.

	Breast cancer		p-value
	No	Yes	
	(N = 1074402, %)	(N = 10461, %)	
^*^Age (years)	58.43 ± 5.8	57.76 ± 5.55	<.0001
Smoking			0.0375
Never	1035039 (96.34)	10035 (95.93)	
Past smoker	11137(1.04)	132 (1.26)	
Current smoker	28226 (2.63)	294 (2.81)	
Alcohol consumption			0.0655
No	924240(86.02)	8916(85.23)	
Mild	143794(13.38)	1481(14.16)	
Heavy	6368 (0.59)	64 (0.61)	
Regular physical activity	213260 (19.85)	2176 (20.8)	0.0152
Hypertension	438793(40.84)	4364(41.72)	0.0696
Diabetes mellitus	82266 (7.66)	877(8.38)	0.0054
Dyslipidemia	365533(34.02)	3733 (35.68)	0.0004
^*^BMI (kg/m^2^)	24.23 ± 3.09	24.49 ± 3.14	<.0001
^*^Cholesterol	209.1 ± 43.65	208.99 ± 42.87	0.7919
^*^LDL	127.99 ± 70.43	128.55 ± 69.55	0.4225
^*^HDL	58.11 ± 34.62	58.31 ± 36.69	0.5615
^**^GGT	20.91(20.88, 20.93)	21.98 (21.73,22.22)	<0.0001

^*^Parameter was indicated as mean +/- SD.

^**^Geometric means(95% C.I).

### Higher Serum GGT Level Increases the Risk of Breast Cancer

Individual data were categorized into quartiles to investigate the association between GGT levels. The GGT ranges of each quartile (Q1-Q4) were displayed in **Table 3**. The normal cut-off value for serum GGT in Korean is 35 IU/L. Only a portion of women in the quartile group 4 were above the normal serum GGT value. The Q1 was set as a reference for further analysis to investigate the relationship between serum GGT level and breast cancer risk.

**Table 3:** Ranges of GGT levels in each quartile.

Quartile	Serum GGT levels (IU/L)
Q1	<14.0
Q2	≥14.0 and <18.0
Q3	≥18.0 and <26.0
Q4	≥26.0

**Figure 2:**
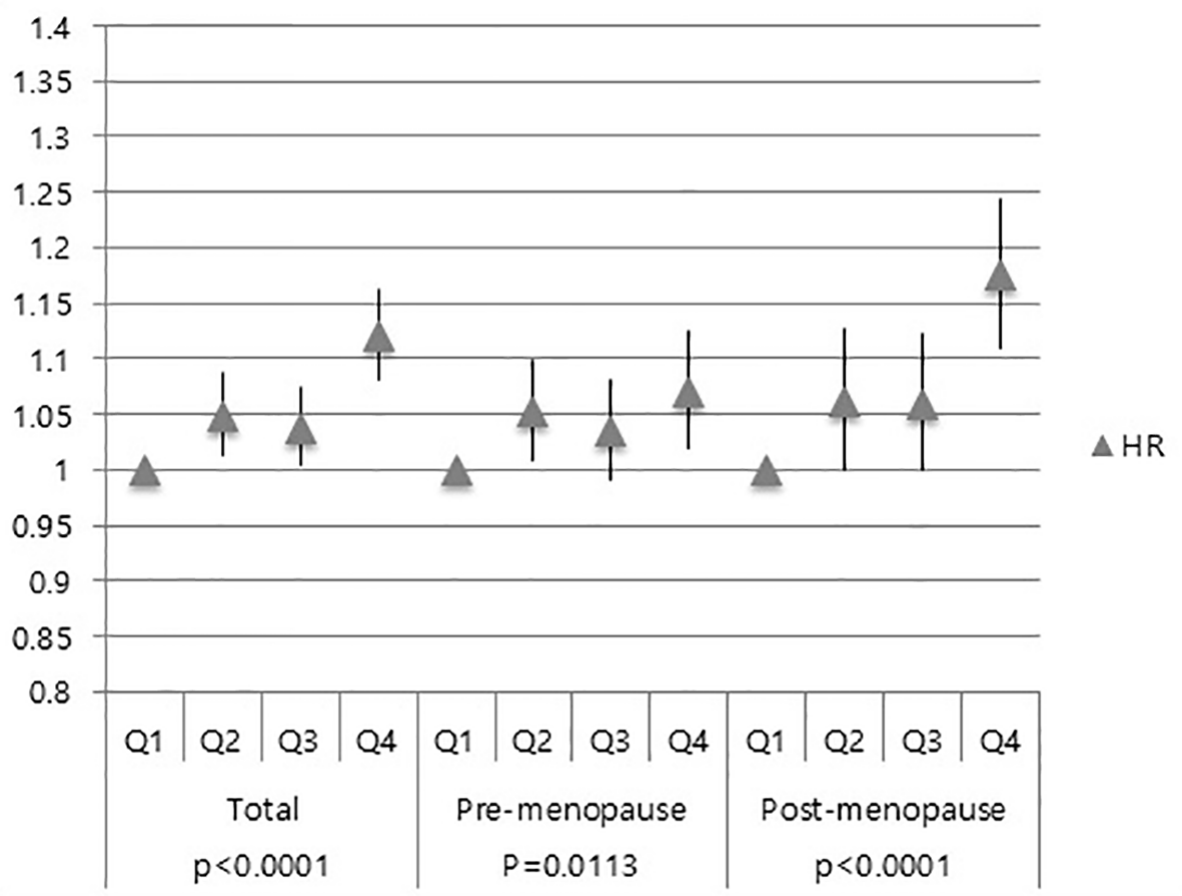
Association of GGT levels with breast cancer risk in total subjects, pre-menopausal women and post-menopausal women.

Overall, women with higher GGT levels (Q4) had an increased risk of developing breast cancer, compared to those with Q1 (HR: 1.120, 95% CI: 1.080–1.162) regardless of the participants’ menopausal status (**Figure 2**). Both Q2 and Q3 groups also showed increased breast cancer risk than the Q1 group (**Figure 2**).

Similar results were also obtained in pre-menopausal subgroup and post-menopausal subgroup (**Figure 2**). Moreover, the Q4 group’s HR for breast cancer was higher in the post-menopausal group (HR: 1.173, 95% CI: 1.107–1.243) than that in the pre-menopausal group (HR: 1.070, 95% CI: 1.019–1.124) (**Figure 2**).

**Figure 3:**
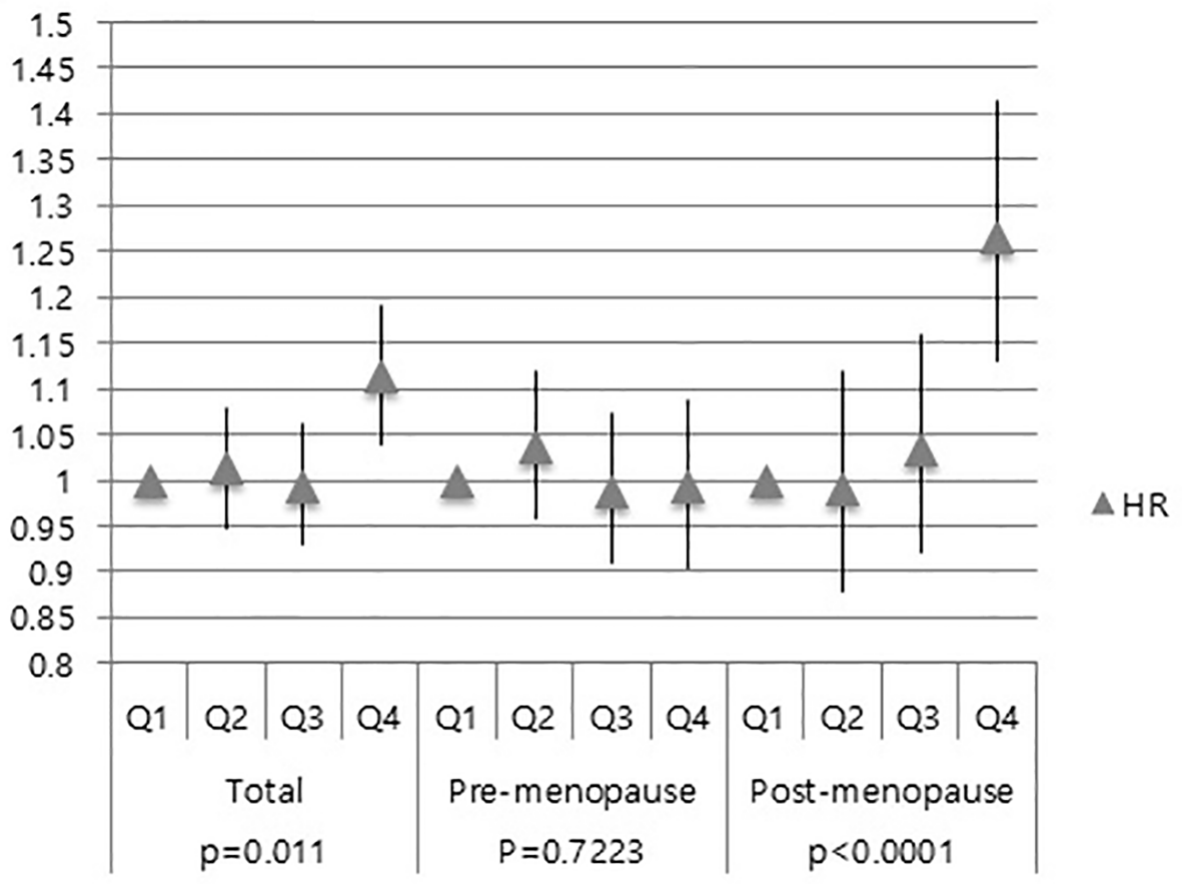
Association of GGT levels with CIS risk in total subjects, pre-menopausal and post-menopausal women.

Further investigation was done to ascertain whether serum GGT level is associated with the development of CIS and invasive breast cancer. Compared to the Q1 group, the Q4 group showed an increased risk of developing CIS (HR: 1.114; 95% CI: 1.040–1.192) (**Figure 3**). However, no difference was observed among the Q2-3 group in the total women group (HR for Q2: 1.006, 95% CI: 0.948–1.08; HR for Q3: 0.994, 95% CI: 0.931–1.062) in the CIS risk (**Figure 3**).

Next, we performed subgroup analysis according to the menopausal status. In the post-menopausal women, the Q4 group exhibited a higher risk of CIS than the Q1 group (HR: 1.266, 95% CI: 1.132–1.416) (**Figure 3**). However, no difference was observed the Q2, Q3, and Q4 group in the pre-menopausal women (HR: 1.035, 95% CI: 0.959–1.119; HR: 0.988, 95% CI: 0.911–1.072; HR: 0.992, 95% CI: 0.905–1.087) (**Figure 3**). These results showed that elevated serum GGT level was associated with a higher risk of developing CIS in post-menopausal women.

Subsequently, the effects of serum GGT levels in post-menopausal women were analyzed by age (**Figure S1**). The breast cancer incidence of Q1 was set as a reference for the analysis. In post-menopausal women, women with elevated serum GGT level (Q4) had a high risk of developing breast cancer at all ages except for women aged between 55 and 60, with the highest risk in women over 65 years (HR:1.272, 95% CI:1.085-1.49) (**Figure S1**).

Additionally, post-menopausal women with elevated serum GGT level (Q4) had a high risk of developing CIS at all ages except for women aged less than 55, also with the highest risk in women over 65 years(HR 1.508, 95% CI 1.084-2.099) (**Figure S2**).

### High Serum GGT Levels Increase Breast Cancer Risk Particularly in Obese Women

**Figure 4:**
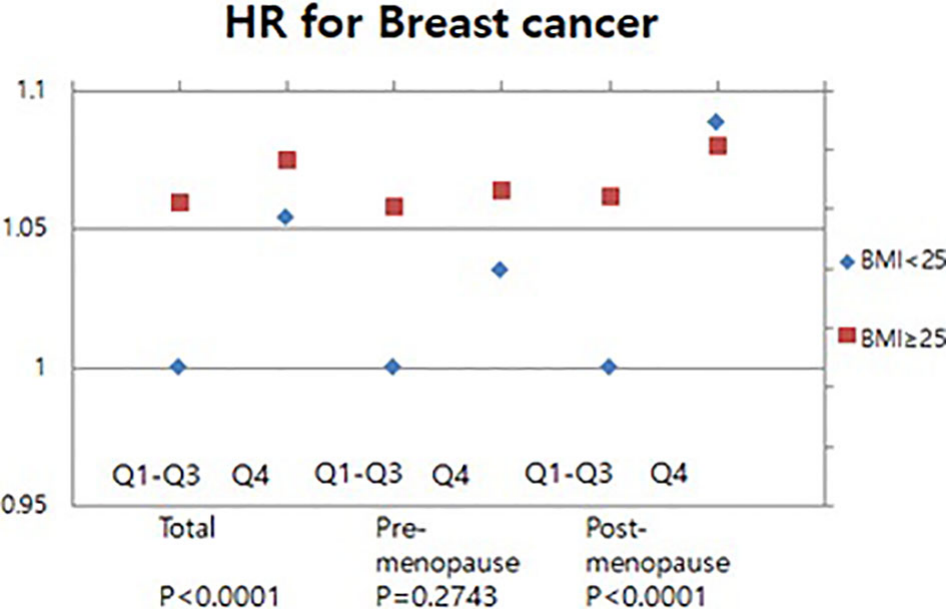
Association of serum GGT levels with breast cancer in pre-and post-menopausal women by BMI.

We conducted the further analysis with additional discussion of the presence of obesity. The Q1-Q3 with BMI less than 25 kg/m^2^ was set as a reference for the analysis. The relationship between serum GGT level and the breast cancer risk in obese and non-obese women was assessed. Our result showed that the breast cancer risk of women with BMI more than 25 kg/m^2^ (HR 1.165, 95% CI 1.103-1.231) was higher than those of women with BMI less than 25 kg/m^2^ (HR 1.054, 95% CI 1.014-1.096) (**Figure 4**). There was no significant association of serum GGT level and breast cancer risks in pre-menopausal women when subcategorized into obese- and non-obese groups (**Figure 4**). However, the subgroup analysis for post-menopausal women revealed that elevated serum GGT level was associated with increased breast cancer risk in obese women (**Figure 4**). HRs for the quartiles Q1-3 was 1.042 (95% CI 0.973-1.115) and HR for the quartiles Q4 was HR 1.214 (95% CI 1.125-1.31) (**Figure 4**).

**Figure 5:**
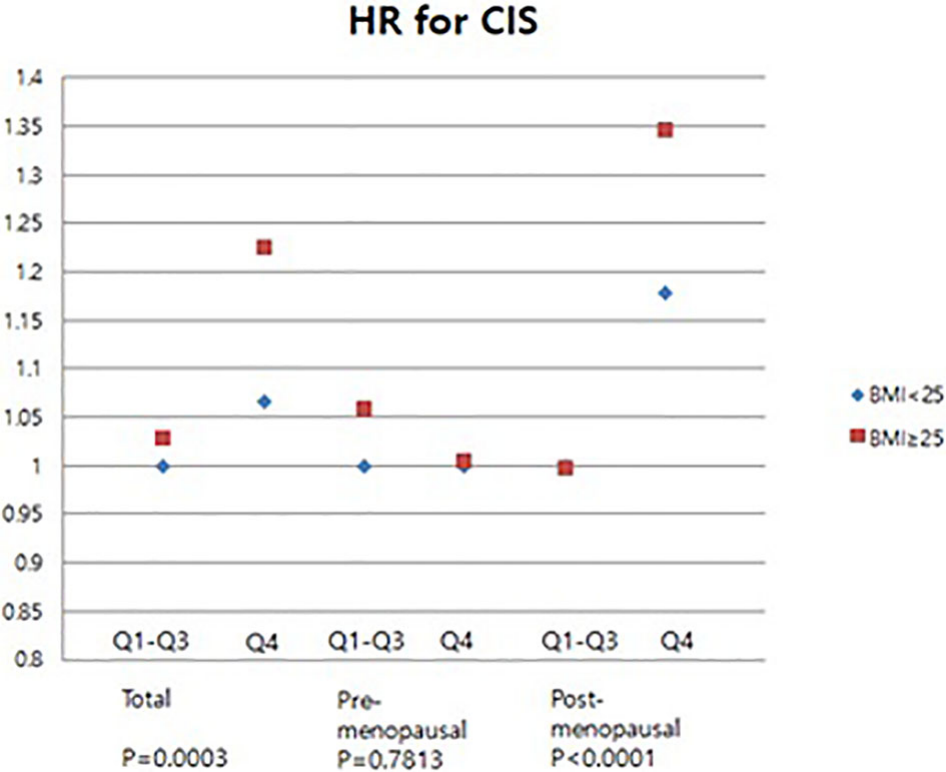
Association of levels of GGT with carcinoma *in situ* of breast in pre- and post-menopausal women by BMI.

Meanwhile, an analysis was also performed about the incidence of CIS (**Figure 5**). In obese women, the risk of CIS was significantly increased in the high GGT level group (HR 1.226, 95% CI 1.101-1.365) (**Figure 5**). No significant association of serum GGT level and CIS risks when pre-menopausal women were subcategorized into obese- and non-obese groups(**Figure 5**). However, subgroup analysis for post-menopausal women revealed that in the Q4 group, the CIS risk was higher in obese women (HR: 1.346; 95% CI: 1.157-1.566) than that in non-obese women (HR: 1.18; 95% CI: 1.059-1.316) (**Figure 5**). These results indicate that high serum GGT level related to the risk of developing breast cancer and CIS particularly in obese women.

## Discussion

In this nationwide cohort study, we suggest serum GGT as a novel biomarker of breast cancer risk. Our results mainly showed that women exhibiting higher serum GGT levels had a high risk of developing breast cancer later according to the menopausal status, obesity status, age and conducted subgroup analyses. Additionally, subgroup analysis was conducted according to the menopausal status, age and obesity status. The subgroup analysis suggests that obese post-menopausal elderly women were more susceptible to breast cancer and CIS with elevated GGT levels.

A previous study analyzing the data from a large cohort in Sweden showed a positive correlation between GGT levels and overall primary cancer risk. The cancer-site specific analysis showed that digestive organ, respiratory system/intra-thoracic organs, female genital organ/breast, urinary organs, and male genital organs cancer risk was significantly elevated in people with high serum level of GGT(>36U/L) (22). Another population-based cohort study in Austria showed that elevated serum GGT(≥72 U/L) increased cancer risk (HR:1.43,95% CI:1.28-1.61). In cancer-site specific analysis, high level of GGT significantly increased the risk for cancer in digestive organs, the respiratory/intra-thoracic organs, female genital organ/breast and lymphoid/hematopoietic cancer (19). Despite that these large cohort studies have been conducted in western countries, large cohort studies were also needed in Asia since breast cancer in Asia has a different epidemiologic characteristics compared to western countries. To the best of our knowledge, this is the first study in a large data set reporting the association between high levels of GGT and breast cancer incidence in Asia.

GGT is a main enzyme related to glutathione metabolism (23). GGT has been reported about involvement of a pro-oxidant activity and its early recognized contributions to cellular antioxidant defenses (13). Pro-oxidants derived from GGT can regulate important redox-sensitive processes and functions of the cell with particular reference to proliferative/apoptotic balance (24). It has obvious and important implications in tumor progression and drug resistance (24). Some recent studies have suggested that the serum GGT level is associated with cancer prognosis. Grim m et al. reported that high pre-therapeutic serum GGT level (> 72 IU/L) was significantly related to the worse overall survival of epithelial ovarian cancer patients and also associated with elevated GGT expression in the epithelial ovarian tissue (25). Another study demonstrated that the pre-treatment elevated serum GGT was associated with low 5-year progression-free survival in endometrial cancer (26). These results are explained by the study that the high expression of GGT in cancer cells is significantly related to drug resistance (27). Through the studies so far, elevated serum GGT levels seem to have an effect on various aspects from cancer development and progression.

We analyzed a large cohort covering almost the entire Korean women aged over 40 years old who were eligible for national breast cancer screening. The median follow-up period of the current study was 8.37 years, which is a relatively long period for monitoring the incidence of cancer. The cut-off value of quartile group4, serum GGT level of 26U/L, is suggested as a new cut-off value for Korean women for predicting the breast cancer risk. In addition, obesity was shown as a co-factor with the high serum GGT level in contributing the increasing breast cancer risk. However, the role of GGT in breast tumorigenesis remains largely uninvestigated. Therefore, future studies elucidating GGT on the breast cancer etiology are warranted.

## Conclusions

To summarize, our study elucidated the association between serum GGT and the risk of developing breast cancer by utilizing a big dataset from the Korean health insurance database. Elevated serum GGT is associated with increased breast cancer incidence particularly in post-menopausal women with obesity. We believe that our study could provide a new insight into the strategy of breast cancer prevention.

## Data Availability Statement

The datasets presented in this study can be found in online repositories. The names of the repository/repositories and accession number(s) can be found below: the Korea National Health Insurance Sharing Service homepage (nhiss.nhis.co.kr).

## Ethics Statement

The studies involving human participants were reviewed and approved by the Institutional Review Board (IRB) of Seoul National University Hospital (IRB number: 2101-003-1184). Written informed consent for participation was not required for this study in accordance with the national legislation and the institutional requirements.

## Author Contributions

AS, WW, SK, YH, IP, JY, HJ, K-DH, and YS: design, collection of data, manuscript, editing, approval of final version, and accountability. All authors contributed to the article and approved the submitted version.

## Funding

This research was supported by BK21 Plus Program of the Department of Agricultural Biotechnology, Seoul National University (Seoul, Korea), a grant of Health Technology R&D Project through the Korea Health Industry Development R&D Project through the Korea Health Industry Development Institute (KHIDI), funded by the Ministry of Health & Welfare, Republic of Korea [grant number: HI16C2037] and a grant from the National R&D Program for Cancer Control, Ministry of Health & Welfare, Republic of Korea [grant number: HA17C0037].

## Conflict of Interest

The authors declare that the research was conducted in the absence of any commercial or financial relationships that could be construed as a potential conflict of interest.

## Supplementary Material

Supplementary Figure 1 Association of GGT levels with breast cancer risk by age in post-menopausal women.

Supplementary Figure 2 Association of GGT levels with carcinoma *in situ* of breast risk by age in post-menopausal women.
