# Supplementary figures and images for: Enhanced Susceptibility to Breast Cancer in Korean Women With Elevated Serum Gamma-Glutamyltransferase Levels: A Nationwide Population-Based Cohort Study
Source: Front Oncol. 2021 May 27;11:668624. doi: 10.3389/fonc.2021.668624 (PMC8191736; doi:10.3389/fonc.2021.668624)

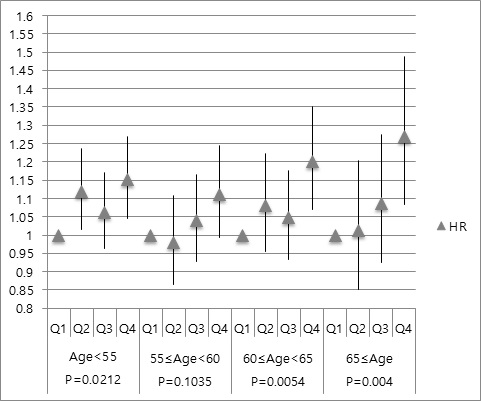

Supplement: Supplementary Figure 1 — Association of GGT levels with breast cancer risk by age in post-menopausal women. [file Image_1.jpeg]

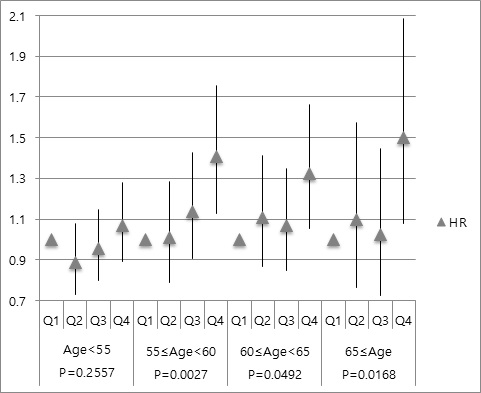

Supplement: Supplementary Figure 2 — Association of GGT levels with carcinoma in situ of breast risk by age in post-menopausal women. [file Image_2.jpeg]
