# Supplementary material for: Enhanced Susceptibility to Breast Cancer in Korean Women With Elevated Serum Gamma-Glutamyltransferase Levels: A Nationwide Population-Based Cohort Study
Source: Front Oncol. 2021 May 27;11:668624. doi: 10.3389/fonc.2021.668624 (PMC8191736; doi:10.3389/fonc.2021.668624)
Supplement: Supplementary file 3 [file DataSheet_1.docx]

**Supplementary Table 1**. Breast cancer and CIS risk in total subjects, premenopausal women and postmenopausal women according to GGT level.

| **RGGT_Q** | **N** | **EVENT** | **DURATION (person-year)** | **RATE** | ^*^**MODEL 1**  **HR** | ^†^**MODEL 2**  **HR** | ^‡^**MODEL 3**  **HR** | ^§^**MODEL 4**  **HR** | **p-value** |
| --- | --- | --- | --- | --- | --- | --- | --- | --- | --- |
|  | **Total** | | | | | | | |  |
| **Breast cancer** |  |  |  |  |  |  |  |  |  |
| Q1 | 559618 | 6898 | 4665350.44 | 1.47856 | 1(Ref.) | 1(Ref.) | 1(Ref.) | 1(Ref.) | <.0001 |
| Q2 | 476912 | 5881 | 3978988.81 | 1.47801 | 1(0.966,1.035) | 1.045 (1.009,1.082) | 1.043 (1.007,1.08) | 1.049 (1.013,1.087) |  |
| Q3 | 532968 | 6260 | 4451903.62 | 1.40614 | 0.952(0.92,0.985) | 1.033(0.998,1.069) | 1.028(0.992,1.064) | 1.038(1.003,1.075) |  |
| Q4 | 497487 | 6142 | 4156619.31 | 1.47764 | 1(0.966,1.035) | 1.115(1.077,1.154) | 1.104(1.064,1.145) | 1.121(1.081,1.162) |  |
| **CIS** |  |  |  |  |  |  |  |  |  |
| Q1 | 559618 | 2070 | 4684887.91 | 0.44185 | 1(Ref.) | 1(Ref.) | 1(Ref.) | 1(Ref.) | 0.0043 |
| Q2 | 476912 | 1649 | 3995682.61 | 0.4127 | 0.934(0.876,0.997) | 0.99(0.928,1.056) | 1.006 (0.942,1.073) | 1.012(0.948,1.08) |  |
| Q3 | 532968 | 1682 | 4469960.48 | 0.37629 | 0.852(0.799,0.909) | 0.95(0.89,1.013) | 0.984(0.922,1.051) | 0.994(0.931,1.062) |  |
| Q4 | 497487 | 1640 | 4174295.66 | 0.39288 | 0.89(0.834,0.949) | 1.028(0.963,1.098) | 1.097(1.025,1.175) | 1.113(1.039,1.192) |  |
|  | **Pre-menopausal** | | | | | | | |  |
| **Breast cancer** |  |  |  |  |  |  |  |  |  |
| Q1 | 337575 | 4965 | 2799330.57 | 1.77364 | 1(Ref.) | 1(Ref.) | 1(Ref.) | 1(Ref.) | 0.0113 |
| Q2 | 239508 | 3669 | 1984724.31 | 1.84862 | 1.042(0.999,1.088) | 1.044(1,1.09) | 1.051(1.007,1.097) | 1.052(1.008,1.098) |  |
| Q3 | 225663 | 3363 | 1870069.44 | 1.79833 | 1.014(0.97,1.059) | 1.018(0.974,1.064) | 1.034(0.989,1.081) | 1.035(0.99,1.082) |  |
| Q4 | 179382 | 2723 | 1486344.74 | 1.83201 | 1.033(0.986,1.082) | 1.041(0.993,1.091) | 1.071(1.019,1.124) | 1.07(1.019,1.124) |  |
| **CIS** |  |  |  |  |  |  |  |  |  |
| Q1 | 337575 | 1556 | 2812965.12 | 0.55315 | 1(Ref.) | 1(Ref.) | 1(Ref.) | 1(Ref.) | 0.7223 |
| Q2 | 239508 | 1109 | 1994601.97 | 0.556 | 1.005(0.931,1.086) | 1.009(0.934,1.09) | 1.035(0.958,1.118) | 1.035(0.959,1.119) |  |
| Q3 | 225663 | 959 | 1879484.97 | 0.51025 | 0.923(0.851,1) | 0.932(0.86,1.01) | 0.987(0.91,1.071) | 0.988(0.911,1.072) |  |
| Q4 | 179382 | 723 | 1494225.39 | 0.48386 | 0.875(0.801,0.956) | 0.892(0.816,0.974) | 0.994(0.907,1.09) | 0.992(0.905,1.087) |  |
|  | **Post-menopausal** | | | | | | | |  |
| Breast cancer |  |  |  |  |  |  |  |  |  |
| Q1 | 222043 | 1933 | 1866019.87 | 1.03589 | 1(Ref.) | 1(Ref.) | 1(Ref.) | 1(Ref.) | <.0001 |
| Q2 | 237404 | 2212 | 1994264.5 | 1.10918 | 1.071(1.007,1.138) | 1.079(1.015,1.147) | 1.065(1.002,1.132) | 1.061(0.999,1.128) |  |
| Q3 | 307305 | 2897 | 2581834.19 | 1.12207 | 1.083(1.023,1.147) | 1.099(1.037,1.164) | 1.065(1.005,1.129) | 1.059(0.999,1.122) |  |
| Q4 | 318105 | 3419 | 2670274.58 | 1.28039 | 1.236(1.169,1.307) | 1.249(1.182,1.321) | 1.185(1.119,1.255) | 1.175(1.109,1.244) |  |
| CIS |  |  |  |  |  |  |  |  |  |
| Q1 | 222043 | 514 | 1871922.8 | 0.27458 | 1(Ref.) | 1(Ref.) | 1(Ref.) | 1(Ref.) | <.0001 |
| Q2 | 237404 | 540 | 2001080.64 | 0.26985 | 0.983(0.871,1.109) | 0.993(0.88,1.121) | 0.995(0.881,1.123) | 0.991(0.878,1.119) |  |
| Q3 | 307305 | 723 | 2590475.52 | 0.2791 | 1.016(0.908,1.138) | 1.036(0.926,1.161) | 1.039(0.927,1.165) | 1.033(0.921,1.158) |  |
| Q4 | 318105 | 917 | 2680070.27 | 0.34216 | 1.246(1.119,1.388) | 1.265(1.135,1.409) | 1.274(1.139,1.424) | 1.264(1.13,1.413) |  |

Abbreviations: HR, hazard ratio; Ref., reference.

^*^Unadjusted.

^†^Adjusted for age.

^‡^Adjusted for age, smoking status, alcohol consumption, physical activity, and diabetes

^§^ Adjusted for age, smoking status, alcohol consumption, physical activity, diabetes, number of parities, and duration of hormone replacement therapy.

**Supplementary Table2.** Breast cancer and CIS risk in total subjects, premenopausal women and postmenopausal women according to GGT level and BMI.

| **RGGT_OBESITY** | **N** | **EVENT** | **DURATION (person-year)** | **RATE** | ^*^**MODEL 1** | ^†^**MODEL 2** | ^‡^**MODEL 3** | ^§^**MODEL 4** | **p-value** |
| --- | --- | --- | --- | --- | --- | --- | --- | --- | --- |
|  |  |  |  |  | **HR** | **HR** | **HR** | **HR** |  |
|  | **TOTAL** | | | | | | | |  |
| **Breast** |  |  |  |  |  |  |  |  |  |
| RGGT_Q =Q1,Q2,Q3 / BMI < 25 | 1148610 | 14159 | 9575643.2 | 1.47865 | 1(Ref.) | 1(Ref.) | 1(Ref.) | 1(Ref.) | <.0001 |
| RGGT_Q =Q1,Q2,Q3 / BMI ≥ 25 | 420888 | 4880 | 3520599.67 | 1.38613 | 0.938(0.908,0.969) | 1.024(0.991,1.059) | 1.024(0.978,1.073) | 1.021(0.975,1.07) |  |
| RGGT_Q = Q4 / BMI < 25 | 269932 | 3250 | 2253356.71 | 1.44229 | 0.976(0.939,1.014) | 1.042(1.003,1.083) | 1.042(1.003,1.083) | 1.054(1.014,1.095) |  |
| RGGT_Q =Q4 / BMI ≥ 25 | 227555 | 2892 | 1903262.6 | 1.5195 | 1.029(0.988,1.071) | 1.162(1.116,1.21) | 1.161(1.099,1.226) | 1.165(1.103,1.231) |  |
| **CIS** |  |  |  |  |  |  |  |  |  |
| RGGT_Q =Q1,Q2,Q3 / BMI < 25 | 1148610 | 4201 | 9615503.88 | 0.4369 | 1(Ref.) | 1(Ref.) | 1(Ref.) | 1(Ref.) | 0.0003 |
| RGGT_Q =Q1,Q2,Q3 / BMI ≥ 25 | 420888 | 1200 | 3535027.13 | 0.33946 | 0.777(0.729,0.829) | 0.871(0.817,0.93) | 1.036(0.946,1.134) | 1.029(0.94,1.127) |  |
| RGGT_Q = Q4 / BMI < 25 | 269932 | 929 | 2262558.84 | 0.4106 | 0.94(0.876,1.009) | 1.025(0.954,1.101) | 1.056(0.982,1.135) | 1.067(0.993,1.147) |  |
| RGGT_Q =Q4 / BMI ≥ 25 | 227555 | 711 | 1911736.82 | 0.37191 | 0.852(0.787,0.922) | 0.999(0.922,1.083) | 1.225(1.101,1.364) | 1.225(1.101,1.364) |  |
|  | **Pre-menopausal** | | | | | | | |  |
| **Breast** |  |  |  |  |  |  |  |  |  |
| RGGT_Q =Q1,Q2,Q3 / BMI < 25 | 631862 | 9566 | 5236627.31 | 1.82675 | 1(Ref.) | 1(Ref.) | 1(Ref.) | 1(Ref.) | 0.2743 |
| RGGT_Q =Q1,Q2,Q3 / BMI ≥ 25 | 170884 | 2431 | 1417497.01 | 1.71499 | 0.939(0.898,0.982) | 0.946(0.905,0.989) | 1.016(0.953,1.082) | 1.01(0.948,1.077) |  |
| RGGT_Q = Q4 / BMI < 25 | 107089 | 1654 | 887120.81 | 1.86446 | 1.021(0.969,1.075) | 1.026(0.974,1.081) | 1.035(0.981,1.091) | 1.035(0.982,1.091) |  |
| RGGT_Q =Q4 / BMI ≥ 25 | 72293 | 1069 | 599223.93 | 1.78397 | 0.977(0.917,1.041) | 0.987(0.926,1.051) | 1.076(0.991,1.168) | 1.065(0.981,1.156) |  |
| **CIS** |  |  |  |  |  |  |  |  |  |
| RGGT_Q =Q1,Q2,Q3 / BMI < 25 | 631862 | 2976 | 5262714.01 | 0.56549 | 1(Ref.) | 1(Ref.) | 1(Ref.) | 1(Ref.) | 0.7813 |
| RGGT_Q =Q1,Q2,Q3 / BMI ≥ 25 | 170884 | 648 | 1424338.04 | 0.45495 | 0.805(0.739,0.876) | 0.819(0.752,0.892) | 1.071(0.95,1.207) | 1.059(0.939,1.194) |  |
| RGGT_Q = Q4 / BMI < 25 | 107089 | 477 | 891763.35 | 0.5349 | 0.946(0.859,1.042) | 0.959(0.87,1.056) | 1(0.907,1.103) | 0.999(0.906,1.102) |  |
| RGGT_Q =Q4 / BMI ≥ 25 | 72293 | 246 | 602462.04 | 0.40832 | 0.723(0.634,0.823) | 0.741(0.65,0.844) | 1.025(0.872,1.205) | 1.006(0.856,1.183) |  |
|  | **Post-menopausal** | | | | | | | |  |
| **Breast** |  |  |  |  |  |  |  |  |  |
| RGGT_Q =Q1,Q2,Q3 / BMI < 25 | 516748 | 4593 | 4339015.9 | 1.05853 | 1(Ref.) | 1(Ref.) | 1(Ref.) | 1(Ref.) | <.0001 |
| RGGT_Q =Q1,Q2,Q3 / BMI ≥ 25 | 250004 | 2449 | 2103102.66 | 1.16447 | 1.101(1.048,1.156) | 1.14(1.085,1.197) | 1.034(0.965,1.107) | 1.042(0.973,1.115) |  |
| RGGT_Q = Q4 / BMI < 25 | 162843 | 1596 | 1366235.9 | 1.16817 | 1.104(1.042,1.168) | 1.105(1.044,1.169) | 1.094(1.033,1.158) | 1.088(1.027,1.152) |  |
| RGGT_Q =Q4 / BMI ≥ 25 | 155262 | 1823 | 1304038.67 | 1.39796 | 1.322(1.252,1.395) | 1.359(1.287,1.435) | 1.213(1.124,1.309) | 1.216(1.126,1.312) |  |
| **CIS** |  |  |  |  |  |  |  |  |  |
| RGGT_Q =Q1,Q2,Q3 / BMI < 25 | 516748 | 1225 | 4352789.87 | 0.28143 | 1(Ref.) | 1(Ref.) | 1(Ref.) | 1(Ref.) | <.0001 |
| RGGT_Q =Q1,Q2,Q3 / BMI ≥ 25 | 250004 | 552 | 2110689.09 | 0.26153 | 0.93(0.841,1.028) | 0.972(0.879,1.076) | 0.992(0.863,1.14) | 0.998(0.869,1.147) |  |
| RGGT_Q = Q4 / BMI < 25 | 162843 | 452 | 1370795.49 | 0.32974 | 1.172(1.052,1.305) | 1.173(1.053,1.307) | 1.184(1.062,1.32) | 1.179(1.058,1.314) |  |
| RGGT_Q =Q4 / BMI ≥ 25 | 155262 | 465 | 1309274.78 | 0.35516 | 1.262(1.135,1.405) | 1.309(1.176,1.457) | 1.344(1.155,1.564) | 1.346(1.157,1.566) |  |

Abbreviations: HR, hazard ratio; Ref., reference.

^*^Unadjusted.

^†^Adjusted for age.

^‡^Adjusted for age, smoking status, alcohol consumption, physical activity, and diabetes

^§^ Adjusted for age, smoking status, alcohol consumption, physical activity, diabetes, number of parities, and duration of hormone replacement therapy.

Supplementary Table3. Breast cancer and CIS risk in postmenopausal women according to GGT level and age.

| **Age Group** | **RGGT_Q** | **N** | **BREAST CANCER** | **DURATION** | **RATE** | ^*^**Model 1** | ^†^**Model 2** | ^‡^**Model 3** | ^§^**Model 4** | **p-value** |
| --- | --- | --- | --- | --- | --- | --- | --- | --- | --- | --- |
| **Post-menopausal** |  |  |  |  |  |  |  |  |  |  |
| 40-54 | Q1 | 77021 | 746 | 643890.47 | 1.15858 | 1(Ref.) | 1(Ref.) | 1(Ref.) | 1(Ref.) | 0.0172 |
|  | Q2 | 75438 | 825 | 629893.39 | 1.30975 | 1.13(1.024,1.248) | 1.129(1.022,1.246) | 1.123(1.017,1.24) | 1.12(1.014,1.237) |  |
|  | Q3 | 90596 | 950 | 756588.12 | 1.25564 | 1.084(0.985,1.193) | 1.081(0.982,1.19) | 1.068(0.97,1.177) | 1.062(0.964,1.17) |  |
|  | Q4 | 95976 | 1102 | 801100.76 | 1.37561 | 1.187(1.082,1.303) | 1.184(1.078,1.299) | 1.159(1.053,1.276) | 1.152(1.046,1.269) |  |
| 55-59 | Q1 | 50673 | 493 | 424785.75 | 1.16059 | 1(Ref.) | 1(Ref.) | 1(Ref.) | 1(Ref.) | 0.0275 |
|  | Q2 | 55654 | 536 | 466382.52 | 1.14927 | 0.99(0.876,1.119) | 0.991(0.877,1.12) | 0.982(0.869,1.11) | 0.979(0.866,1.107) |  |
|  | Q3 | 73306 | 761 | 614157.58 | 1.2391 | 1.068(0.953,1.196) | 1.069(0.954,1.197) | 1.045(0.932,1.171) | 1.039(0.927,1.165) |  |
|  | Q4 | 78767 | 893 | 659565.77 | 1.35392 | 1.167(1.045,1.302) | 1.168(1.046,1.303) | 1.122(1.001,1.257) | 1.112(0.992,1.245) |  |
| 60-64 | Q1 | 55792 | 459 | 470681.67 | 0.97518 | 1(Ref.) | 1(Ref.) | 1(Ref.) | 1(Ref.) | 0.003 |
|  | Q2 | 63075 | 573 | 531652.39 | 1.07777 | 1.105(0.977,1.249) | 1.106(0.978,1.25) | 1.087(0.961,1.229) | 1.081(0.956,1.223) |  |
|  | Q3 | 84954 | 764 | 716206.1 | 1.06673 | 1.094(0.974,1.228) | 1.095(0.975,1.229) | 1.055(0.938,1.186) | 1.047(0.932,1.177) |  |
|  | Q4 | 86545 | 917 | 728893.52 | 1.25807 | 1.29(1.153,1.443) | 1.29(1.154,1.443) | 1.215(1.082,1.364) | 1.202(1.071,1.35) |  |
| 65-69 | Q1 | 38557 | 235 | 326661.98 | 0.7194 | 1(Ref.) | 1(Ref.) | 1(Ref.) | 1(Ref.) | 0.0008 |
|  | Q2 | 43237 | 278 | 366336.2 | 0.75887 | 1.055(0.887,1.255) | 1.054(0.886,1.254) | 1.019(0.856,1.213) | 1.013(0.851,1.205) |  |
|  | Q3 | 58449 | 422 | 494882.38 | 0.85273 | 1.185(1.011,1.39) | 1.185(1.01,1.39) | 1.1(0.936,1.291) | 1.087(0.926,1.276) |  |
|  | Q4 | 56817 | 507 | 480714.53 | 1.05468 | 1.466(1.256,1.711) | 1.463(1.253,1.707) | 1.292(1.103,1.514) | 1.269(1.083,1.487) |  |
| **Age Group** | **RGGT_Q** | **N** | **CIS** | **DURATION** | **RATE** | ^*^**Model 1** | ^†^**Model 2** | **Model 3** | **Model 4** | **p-value** |
| **Post-menopausal** |  |  |  |  |  |  |  |  |  |  |
| 40-54 | Q1 | 77021 | 222 | 646026.01 | 0.34364 | 1(Ref.) | 1(Ref.) | 1(Ref.) | 1(Ref.) | 0.3388 |
|  | Q2 | 75438 | 192 | 632433.47 | 0.30359 | 0.883(0.728,1.072) | 0.885(0.73,1.074) | 0.889(0.733,1.079) | 0.887(0.731,1.077) |  |
|  | Q3 | 90596 | 246 | 759291.65 | 0.32399 | 0.943(0.786,1.13) | 0.946(0.789,1.134) | 0.961(0.8,1.154) | 0.956(0.796,1.148) |  |
|  | Q4 | 95976 | 285 | 804286.27 | 0.35435 | 1.032(0.866,1.229) | 1.036(0.869,1.235) | 1.074(0.895,1.288) | 1.067(0.89,1.28) |  |
| 55-59 | Q1 | 50673 | 123 | 426300.76 | 0.28853 | 1(Ref.) | 1(Ref.) | 1(Ref.) | 1(Ref.) | 0.0005 |
|  | Q2 | 55654 | 135 | 467995.4 | 0.28846 | 1(0.783,1.276) | 1.001(0.784,1.278) | 1.01(0.791,1.29) | 1.008(0.789,1.287) |  |
|  | Q3 | 73306 | 199 | 616358.35 | 0.32286 | 1.119(0.894,1.401) | 1.123(0.897,1.406) | 1.142(0.91,1.432) | 1.138(0.907,1.428) |  |
|  | Q4 | 78767 | 263 | 661963.7 | 0.3973 | 1.377(1.112,1.706) | 1.38(1.114,1.709) | 1.418(1.137,1.769) | 1.41(1.13,1.758) |  |
| 60-64 | Q1 | 55792 | 117 | 472149.72 | 0.2478 | 1(Ref.) | 1(Ref.) | 1(Ref.) | 1(Ref.) | 0.0178 |
|  | Q2 | 63075 | 147 | 533376.33 | 0.2756 | 1.112(0.872,1.418) | 1.113(0.873,1.419) | 1.113(0.873,1.419) | 1.106(0.867,1.411) |  |
|  | Q3 | 84954 | 192 | 718530.23 | 0.26721 | 1.078(0.857,1.357) | 1.08(0.858,1.359) | 1.078(0.855,1.36) | 1.07(0.848,1.349) |  |
|  | Q4 | 86545 | 243 | 731591.8 | 0.33215 | 1.34(1.075,1.671) | 1.341(1.075,1.672) | 1.34(1.067,1.682) | 1.324(1.055,1.662) |  |
| 65-69 | Q1 | 38557 | 52 | 327446.31 | 0.1588 | 1(Ref.) | 1(Ref.) | 1(Ref.) | 1(Ref.) | 0.0134 |
|  | Q2 | 43237 | 66 | 367275.44 | 0.1797 | 1.131(0.787,1.627) | 1.13(0.786,1.625) | 1.102(0.766,1.586) | 1.096(0.762,1.577) |  |
|  | Q3 | 58449 | 86 | 496295.3 | 0.17328 | 1.091(0.773,1.539) | 1.09(0.773,1.538) | 1.035(0.732,1.463) | 1.024(0.724,1.448) |  |
|  | Q4 | 56817 | 126 | 482228.49 | 0.26129 | 1.645(1.191,2.272) | 1.638(1.186,2.262) | 1.522(1.093,2.118) | 1.5(1.077,2.087) |  |

Abbreviations: BMI, body mass index; HR, hazard ratio; Ref., reference.

^*^Unadjusted.

^†^Adjusted for age.

^‡^Adjusted for age, smoking status, alcohol consumption, physical activity, and diabetes

^§^ Adjusted for age, smoking status, alcohol consumption, physical activity, diabetes, number of parities, and duration of hormone replacement therapy.
